# Report of Cases Treated in the Philadelphia Dispensary during the Month of May, 1839

**Published:** 1839-06-22

**Authors:** 


*Report of Cases treated in the Philadelphia Dispensary during the month of May*, 1839.

In the North-Western District, Dr. Evans has treated eleven cases; of which ten resulted favourably, and one died. The latter, a child two years of age, attacked with measles while under treatment for an acute gastritis. The eruption was never perfectly developed, and the patient died on the 8th day; the *immediate* cause of death being, apparently, congestion of the lungs.

In the North-Middle District, Dr. Boyer has treated twenty-two cases; of these, twenty-one recovered, and one died. The latter, an infant, died of pertussis, which had been suffered to remain without medical treatment for three weeks. Besides the above, Dr. B. has prescribed for seventy patients at the Dispensary; of whom 38 were males and 32 females. Most of these cases have been discharged, either cured or temporarily relieved—a few still continue under treatment.

In the North-Eastern District, Dr. Patterson has treated thirty-two cases; of these, twenty-eight have been cured, three relieved, and one sent to the Almshouse. In addition to these, he has eleven cases still under treatment—all chronic.

In the South-Eastern District, Dr. Knight has treated twenty-six cases; of these, nineteen have been cured, one died, one discharged, and five remain under treatment. Three cases of measles occurred, terminating favourably. One, an adult of temperate habits, in whom, though considerably advanced in age, the course of the disease was not more violent than in the other two cases, of which both were children under two years of age.

In the South-Middle District, Dr. Berkeley has treated sixteen cases; all of which have terminated favourably. One of these, a girl, (ten years of age, coloured,) had suffered from a slight catarrh for a few days; to which no attention was paid, till a remarkable change in her voice induced her parents to ask medical advice. Upon examining the throat, the fauces were found so much swollen as nearly to close the entrance into the œsophagus; while the uvula was thrown forward against the roof of the mouth, much enlarged, and sloughing at its extremity.

The girl complained of no pain whatever, and seemed to suffer only from an inability to swallow any thing but fluids. She was directed to use stimulating gargles, till there was a return of sensation in the parts, and the uvula was touched four or five times a day with a solution of nitrate of silver, (iiij. gr. to the oz.) On the third day a considerable portion of the uvula was thrown off—the swelling of the fauces had been much reduced; and the sensibility of the whole surface so far restored as to render the use of stimulants no longer necessary. A mild astringent gargle was then directed, which was continued till the eighth day; when the patient was discharged, cured; no symptoms of the affection remaining, except a slight difficulty in pronouncing a few words, in which there were many consonants.

